# Where Wolves Kill Moose: The Influence of Prey Life History Dynamics on the Landscape Ecology of Predation

**DOI:** 10.1371/journal.pone.0091414

**Published:** 2014-03-12

**Authors:** Robert A. Montgomery, John A. Vucetich, Gary J. Roloff, Joseph K. Bump, Rolf O. Peterson

**Affiliations:** 1 Department of Fisheries and Wildlife, Michigan State University, East Lansing, Michigan, United States of America; 2 School of Forest Resources and Environmental Science, Michigan Technological University, Houghton, Michigan, United States of America; University of Lleida, Spain

## Abstract

The landscape ecology of predation is well studied and known to be influenced by habitat heterogeneity. Little attention has been given to how the influence of habitat heterogeneity on the landscape ecology of predation might be modulated by life history dynamics of prey in mammalian systems. We demonstrate how life history dynamics of moose (*Alces alces*) contribute to landscape patterns in predation by wolves (*Canis lupus*) in Isle Royale National Park, Lake Superior, USA. We use pattern analysis and kernel density estimates of moose kill sites to demonstrate that moose in senescent condition and moose in prime condition tend to be wolf-killed in different regions of Isle Royale in winter. Predation on senescent moose was clustered in one kill zone in the northeast portion of the island, whereas predation on prime moose was clustered in 13 separate kill zones distributed throughout the full extent of the island. Moreover, the probability of kill occurrence for senescent moose, in comparison to prime moose, increased in high elevation habitat with patches of dense coniferous trees. These differences can be attributed, at least in part, to senescent moose being more vulnerable to predation and making different risk-sensitive habitat decisions than prime moose. Landscape patterns emerging from prey life history dynamics and habitat heterogeneity have been observed in the predation ecology of fish and insects, but this is the first mammalian system for which such observations have been made.

## Introduction

Spatial variation in predation pressure is a fundamental feature of predation ecology. Such variation has been observed across a variety of spatial scales [1]–[3] and can result from climatic conditions [4], the effect of habitat heterogeneity on predation risk [5], and from anthropogenic habitat disturbance [6]–[8]. Landscape patterns of predation are also attributable to complex prey life histories [9],[10]. For instance, intricate landscape patterns in predation are apparent for some insects, amphibians, and marine fish as a result of complicated life history strategies, such as individuals relying on different habitats during various stages of their life cycle (e.g., [9],[11]). Within mammalian systems predators are often depicted as causal agents influencing spatial patterns regulating prey abundance and mediating herbivory [5],[12]–[14]. These material exchanges between predators, prey, and the landscape have important implications for prey population stability, trophic dynamics, and ecosystem function [15]–[18]. However, given that life history stages are not so pronounced in mammals, they would seem not to have an important influence on landscape patterns in predation.

Recent evidence, derived from examination of predator-killed carcasses in mammalian systems however, suggests otherwise. For instance, wolf (*Canis lupus*) predation influences the distribution of moose (*Alces alces*) carcasses where subsequent decomposition increases soil nutrient cycling and corresponding plant growth [19]. In the same study system (Isle Royale National Park, Lake Superior, USA), wolf-killed moose exhibit age-specific variation in habitat use demonstrating that moose habitat decisions also affect the distribution of wolf-moose interactions [20]. Thus, examination of predator-killed carcasses provides insight into the hunting ecology of predators and the habitat decisions of their prey (see [19],[21]–[25]). What remains unknown is whether life history dynamics of prey contribute to landscape patterns of predation in mammalian systems, as has been observed in non-mammalian systems.

Wolves are highly selective predators that disproportionately kill prey in vulnerable age and condition classes [26]–[29]. Thereby, the age-specific variation in habitat decisions of Isle Royale moose is attributed to risk-sensitive foraging behavior as senescent-aged moose are more vulnerable to wolf predation than are prime-aged moose [20]. This previous research did not evaluate the specific habitat features associated with the locations where wolves kill moose by life history stage, nor did that research assess how moose life history dynamics might generate broad landscape patterns in predation ecology. The objectives of our work herein are to investigate these processes.

Variation in habitat features should be an important component of sites where wolves kill moose by life history stage. For instance, ungulates modulate predation risk through fission-fusion herding, long distance migrations, and the selection of habitat features that provide refugia [30]–[34]. Species with solitary life history strategies that do not migrate (e.g., Isle Royale moose) should be expected to depend greatly on landscape structure to manage predation risk. Risk-sensitive habitat decisions of Isle Royale moose include selection of habitat with patches of dense conifer trees that reduce detection and provide structural protection from predation [26]. On Isle Royale, the spatial configuration of winter predation risk is well understood. For example, habitats along the Lake Superior shoreline tend to be riskier for moose because they are visited more frequently by packs of widely ranging cursorial wolves [20]. However, shoreline habitats tend to have improved foraging opportunities for moose and to be lower in elevation. Inland habitats tend to be less frequented by wolves, higher in elevation, and include forests with varying amounts of conifer trees. For these reasons, we hypothesize that moose with increased vulnerability to predation (those that are senescent) will make specific habitat decisions for areas of the island that have reduced predation risk. Conversely, we hypothesize that moose in prime condition, that are comparatively less vulnerable to predation, will make more general decisions for habitat with improved forage potential. Within that context, we pursue two primary research questions; *i*) Does the pattern of kill sites for moose in senescent condition exhibit greater spatial clustering in specific regions of Isle Royale when compared to the pattern of kill sites for moose in prime condition? *ii*) Does the predicted probability of kill occurrence for senescent moose increase in inland habitats characterized by patches of conifer tree cover and high elevation when compared to the predicted probability of kill occurrence for prime moose?

## Materials and Methods

Isle Royale National Park (544 km^2^) is located in Lake Superior, USA (48°N, 89°W). The island is essentially a single-predator single-prey system [35] that is largely closed to moose and wolf immigration and emigration [36]. The heterogeneity of vegetation structure across the island is associated with geology and soil formation. Specifically, receding Pleistocene glaciers deposited till allowing for deeper soils to form on the gentle topography of the western portion of Isle Royale [37]–[38]. On the eastern portion of Isle Royale however, this glacial scouring exposed bedrock which lead to shallower soils (typically <30 cm).

Between 2000 and 2008 we located carcasses of moose killed by wolves during winter. We had permission to collect and use the samples for this work which complies with the current Michigan Technological University Institutional Animal Care and Use Committee guidelines, which are guided by the US federal regulations and ethical principles, intended to ensure the humane care and use of animals in research. Use of these data was additionally approved by the Michigan State University Institutional Animal Care and Use Committee. Most carcasses were located during winter aerial surveys (spanning a 7-week period from January – February of each year) designed to monitor wolf movement across the island. Some additional carcasses were also located during summer ground surveys that involved extensive off-trail searching. Because most carcasses in this sample were located from light fixed-wing aircraft in conjunction with estimating wolf kill rate, few moose that died during each field season were missed [39]. During the study period the moose population was typically comprised of 700 to 1100 individuals with the wolf population ranging between 17 and 30 individuals. The population dynamics of moose are negatively affected by winter severity [40] and predation risk [41]–[43]. During most winters wolf predation accounts for more than 80% of moose deaths (e.g., [44]), and the mean annual predation rate among moose (>9 months of age) is 9.9% [43]. For carcasses discovered in winter, necropsies were conducted after wolves finished feeding on a carcass and left the area, typically within 7 days of the moose's kill event [43].

Necropsies included inferring the cause of death from field sign (e.g., blood on snow and trees, signs of a chase, and signs of struggle including broken branches). For carcasses discovered during summer ground surveys, season of death was estimated from field observations including degree of decomposition and the presence of adult ticks which exist only in winter or early spring. We recorded age of the moose at time of death by counting annual cementum lines in teeth [45]. Juvenile moose (≤1 year-old) were excluded from the analysis because the location of their carcasses are largely determined by the habitat use of their mothers. Moreover, it is reasonable to assume that adult cow moose in our sample did not have calves at the time of their death, because: *i*) calves are easier to kill than their mothers, *ii*) a motherless calf is easy for wolves to kill, and *iii*) it is rare for wolves on Isle Royale to kill a cow and a calf at the same time. We also assessed two senescent-associated pathologies, osteoarthritis [46] and periodontal disease [26], as being absent, mild, moderate, or severe in the moose carcasses.

We classified moose as being in senescent condition if they exhibited signs of senescent-associated pathologies [46]. Among the moose carcasses necropsied between 1959 and 2008 (N = 2,652) the mean age of moose without pathologies was 3.9 years while the mean age of moose with pathologies was 12.0 years (unpublished data). Thereby, we considered moose with moderate to severe osteoarthritis or periodontal disease as senescent, and prime otherwise. Senescent moose, because of their age and pathologic condition, are particularly vulnerable to wolf predation [26],[46].

### Pattern analysis

To determine if the kill sites for senescent moose tended to be more clustered when compared to prime moose we evaluated the pattern in the distribution of wolf-killed carcasses for these different kinds of moose using the Ripley's K function [47],[48]. We calculated this function across the range of distances separating carcasses within Isle Royale. This function quantifies the point pattern intensity within circular distance bands separated by 1 km, in this case. We present the linear form of the K statistic which is known as Ripley's L-function [49];
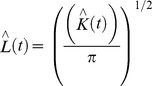
where 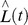 is zero or a completely spatial random (CSR) pattern, under an assumed Poisson process. Thereby, positive 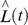 values indicate spatial clustering in the point pattern and negative values indicate spatial dispersion [49]. We compared 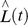 values for prime and senescent moose and performed 99 Monte Carlo simulations to produce confidence envelopes about the estimates. In this fashion we evaluated significant departure from a CSR pattern at the α = 0.01 level.

If significant clustering in kill sites was apparent, our next objective was to evaluate whether the probability of kill occurrence for senescent moose increased in specific areas of the island when compared to prime moose kill occurrence. To facilitate this comparison, we developed kernel density estimates (KDE) of the kill sites for prime and senescent moose. We coded these KDEs in R (R version 2.10.0, <www.cran.r-project.org>, accessed 1 Dec 2010) using least-squares cross-validation [50]. This method provides robust bandwidth estimates, particularly when there are no repeat locations [51]. Resultant data layers were two evaluation surfaces which we converted to raster, based on the height of the KDE, in ArcMap 10.0 (Environmental Systems Research Institute, Redlands, CA). The height of the KDE is equivalent to the probability of kill occurrence.

As is the case for any statistical distribution, the tails of a KDE are more prone to estimation error. For this reason, we used a Bayesian framework to identify the KDE percentile that best represented the divide between core and non-core portions of the KDEs [52]. This Bayesian framework utilizes functions within several R libraries (including MASS, spatstat, splancs, and MCMCpack; [53]–[55]) to evaluate the locational distribution in relation to a CSR pattern [56]. We iterated this process 10,000 times in a Monte Carlo simulation to test for departures from the CSR pattern. The model converges at the density percentile that defines the KDE's core area [52]. Hereafter we refer to these core areas as kill zones.

### Habitat features

Though our database includes wolf-killed moose collected over a 50-year period, we restricted our analysis of the habitat features at kill sites to the period between 2000 and 2008. This period corresponds to the temporal vintage of geospatial data characterizing habitat features of Isle Royale which function as predictor covariates in our regression models. Forest succession on Isle Royale means that modern vegetation maps poorly characterize vegetation patterns in previous decades.

We collated data on a suite of habitat features describing the wolf-moose system of Isle Royale at a resolution of 30 m. Two of these habitat features (canopy cover and conifer cover) were derived from the U.S. Geologic Survey's National Land Cover Database (NLCD; [57]), which is based on data remotely sensed between 2001 and 2006, corresponding to the study period. Proportion canopy cover represented the proportion of each 30 m cell that was covered by canopy while proportion conifer represented the proportion of each cell that was covered with a forest type dominated by conifer trees (i.e., evergreen forest, mixed forest, and palustrine forested wetland). The canopy cover layer has an estimated accuracy >81% [58], and the conifer cover layer has a forest-type classification accuracy of >91% (NLCD 2006 metadata, <http://www.csc.noaa.gov/crs/lca>, accessed on 1 Dec 2010). We used the National Elevation Dataset to portray elevation (in meters) and calculate slope (in degrees). We also assessed distance to Lake Superior shore and distance to nearest inland lake (in meters). Habitats close to the Isle Royale shore tend to be characterized by a greater distribution of preferred conifer species for moose forage (e.g., *A. balsamea*, *Picea glauca*, and *Thuja occidentalis*; [59]–[61]) and greater habitat use by wolves [20]. Thus, shoreline habitat of Isle Royale represents better forage quality and increased predation risk for Isle Royale moose [20]. Because ungulates are vulnerable to wolf predation on inland ice [62] we modeled the distance to frozen inland lakes to account for additional areas of the island with potentially increased predation risk.

### Multiple linear regression

To evaluate whether the probability of kill occurrence for senescent and prime moose was characterized by different habitat features, we built spatially-explicit multiple linear regression models for each type of moose by quantifying habitat in an area around each carcass location of 0.79 ha (equivalent to a 100 m diameter buffer). This scale corresponds to the spatial extent of daily moose movements (i.e., 10′s to 100′s of m; [63],[64]) and to the distances that wolves chase moose before killing them (average<100 m; [65]). Thus, this area likely represents the habitat used by moose in the time period directly before they were killed by wolves. Prior to model fitting, we standardized the habitat features (mean value of 0 and a standard deviation of 1) to allow for comparison of model results between prime and senescent moose.

The models that we constructed in SAS (version 9.2, Cary, NC) took the form; 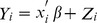
where *Y_i_* is the percentile of the KDE at the *i*th kill site, 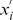 are the observed values of the habitat features at the *i*th kill site, β is a vector of the regression parameters, and 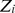 is the random error term with a spatially autocorrelated covariance structure to account for dependencies in habitat features among sites (page 218, [66]). We examined model residuals and found that they were homoscedastic and did not deviate from a normal distribution. We compared habitat features associated with prime and senescent moose kill sites by graphing the regression coefficients produced from the respective models.

## Results

**Figure 1:**
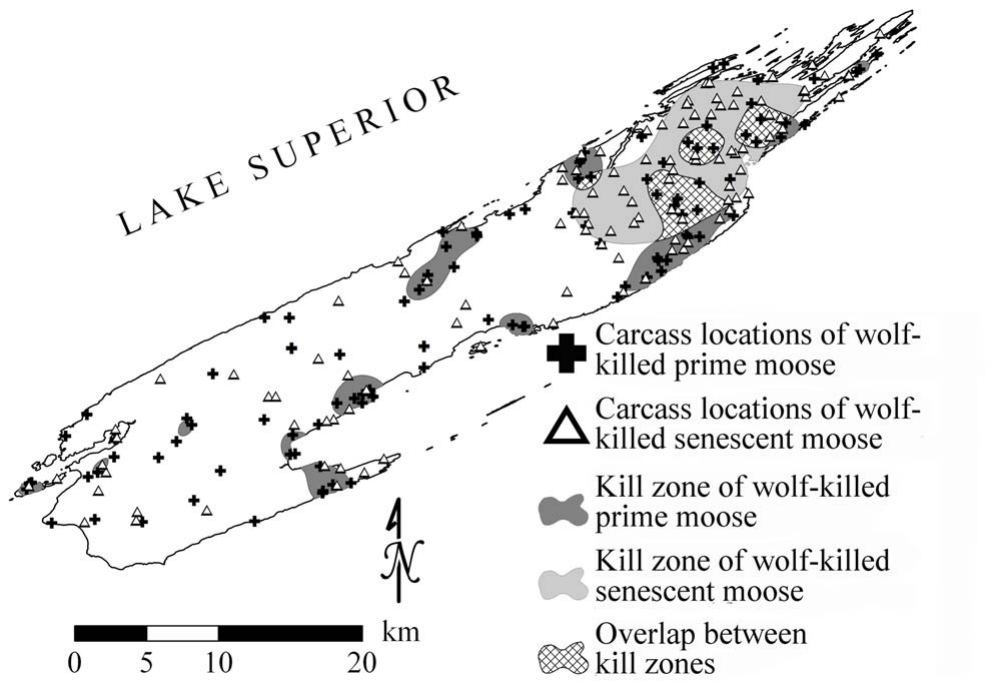
Carcass locations and kill zones, resulting from the core estimation of the kernel density estimates, for prime and senescent moose killed by wolves in Isle Royale National Park, Lake Superior, USA, 2000–2008.

**Figure 2:**
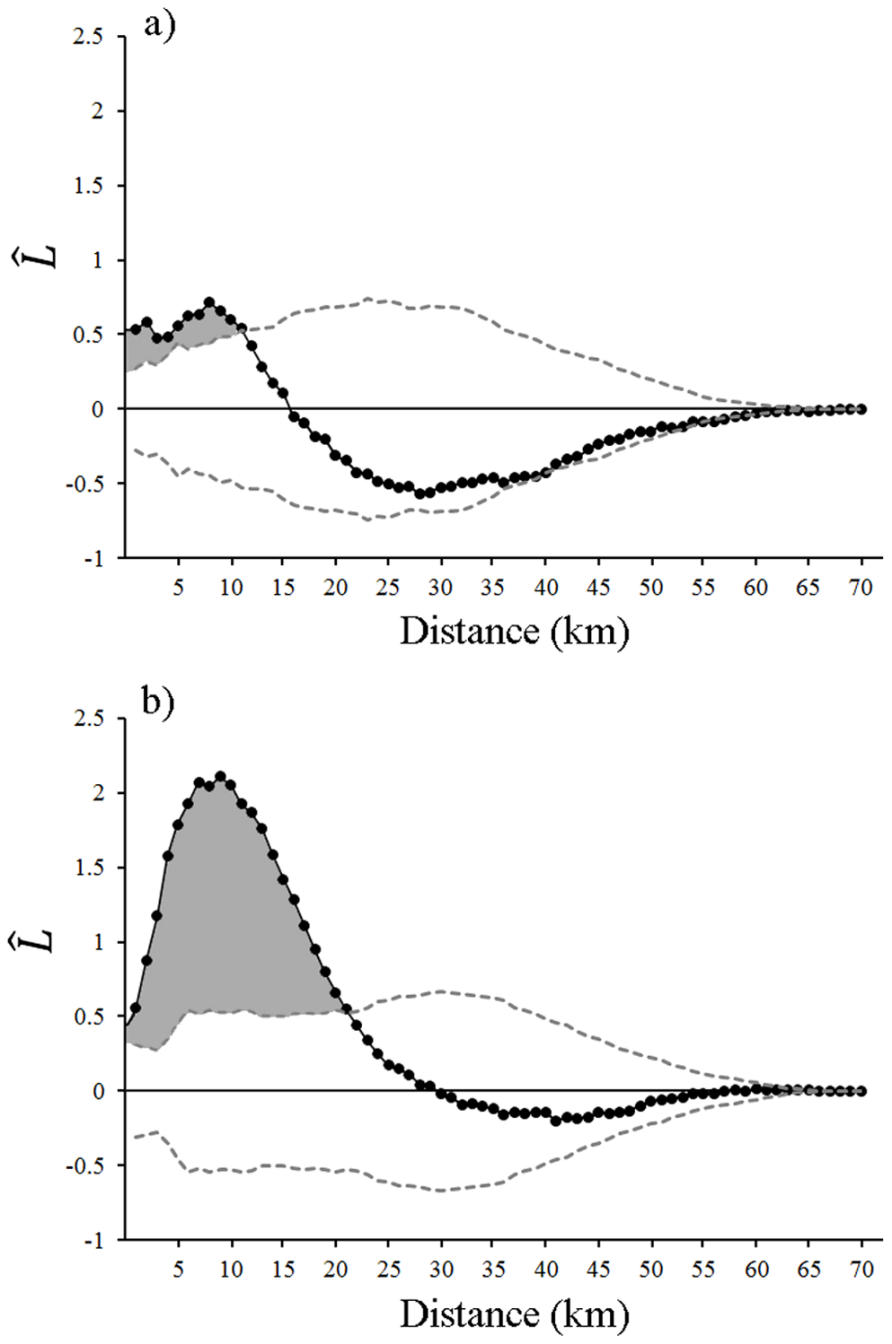
The observed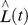 values (plotted with black circles and black line) in relation to distance (km) between locations of a) prime moose and b) senescent moose carcasses in Isle Royale National Park, Lake Superior, USA, 2000–2008. Expected L(t) values are represented by the horizontal line at 0, 99% confidence intervals are depicted with the hatched lines, and significant spatial clustering of carcass locations at the α = 0.01 level is represented by the grey shading.

From 2000–2008 we identified 215 winter wolf-killed moose carcasses across Isle Royale. The divide by life history stage was approximately equal with 106 prime moose and 109 senescent moose (Fig. 1). Ripley's K analysis revealed significant (*P*<0.01) spatial clustering in prime (Fig. 2a) and senescent (Fig. 2b) moose carcass locations and neither distribution was significantly dispersed. However, spatial clustering was more pronounced for senescent moose (Fig. 2b). Senescent moose carcasses were significantly clustered at distances up to 22 km (Fig. 2b). Comparatively, prime moose carcasses were marginally clustered at distances up to 10 km and did not significantly differ from a CSR pattern thereafter (Fig. 2a). Spatial clustering in these data, specifically for senescent moose, validated the use of KDEs to define and compare the core distributions (kill zones) of wolf-killed prime and senescent moose.

Bayesian analysis indicated that the kill zone of the senescent moose KDE corresponded to the top 30% of the distribution (Fig. 1). For comparative purposes, we considered the top 30% of the KDE as the kill zone for prime moose. The extent and location of kill zones differed between prime and senescent moose. The kill zone for senescent moose was a single contiguous polygon covering 102 km^2^ while the kill zone for prime moose was thirteen disjoint polygons totaling 84 km^2^ (Fig. 1). The prime moose kill zones were largely confined to near-shore areas of the island while the senescent moose kill zone was centered in the interior of the northeast portion of the island (Fig. 1). Taken together, the prime and senescent moose kill zones cover just 34% of Isle Royale's area, yet 48% of the prime and senescent moose were wolf-killed within their respective kill zones. This observation, in addition to the Ripley's K assessment, demonstrates that the distribution of wolf-killed moose within the kill zones was higher than what would be expected if kills were distributed randomly throughout the island (χ^2^ = 17.19, *P* = 3.39×10^−5^).

**Figure 3:**
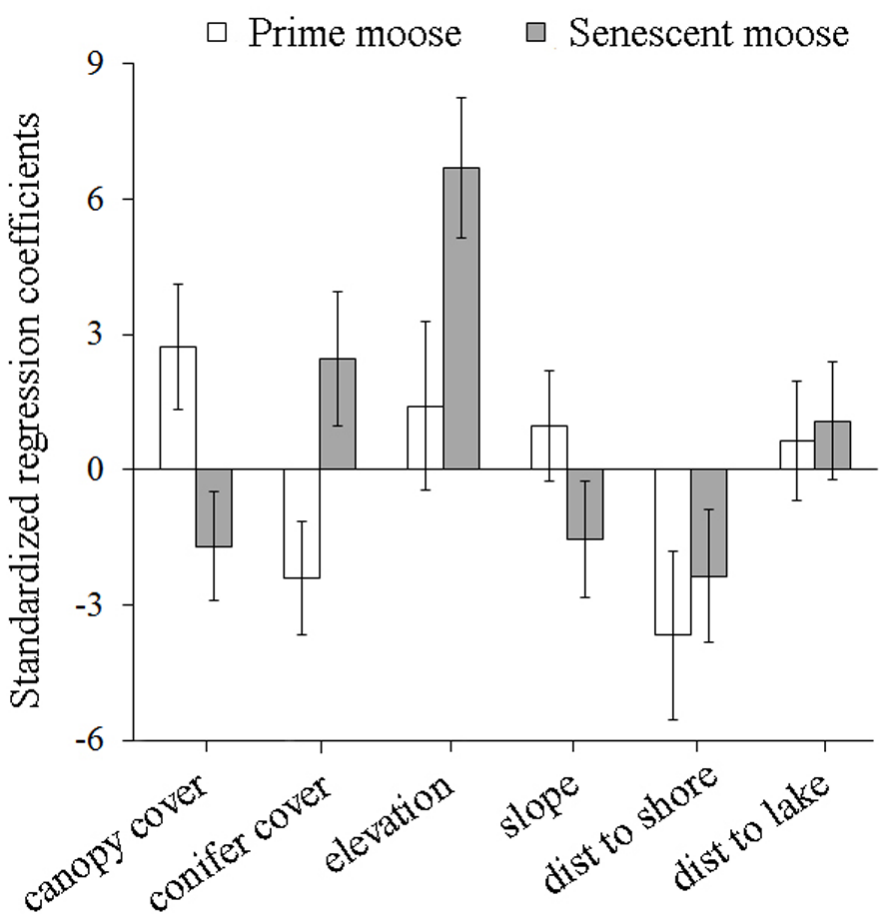
Standardized regression coefficients with standard error bars from the multiple linear regression model describing habitat features at prime and senescent moose kill sites in Isle Royale National Park, Lake Superior, USA, 2000–2008. The response variable for this model was the percentile of the kernel density estimate (see Fig. 1) at each site where a moose was wolf-killed which depicts the probability of kill occurrence. Distance to shore refers to the Lake Superior shoreline, and distance to lake refers to inland lakes within Isle Royale.

Differences in the distribution and extent of kill zones for prime and senescent moose were associated with differences in habitat features. As the probability of kill occurrence for prime moose increased, habitat at kill sites was characterized by greater canopy cover (β = 2.73) and less conifer cover (β = −2.41; Fig. 3). By contrast, habitat at kill sites where senescent moose were more likely to be killed was characterized by higher elevation (β = 6.70), with patches of less canopy cover (β = −1.70) and more conifer cover (β = 2.46; Fig. 3). The relationships between the probability of wolf-killed moose occurrence and both canopy cover and conifer cover were completely opposing by moose life history stage (Fig. 3). Interpretation of the magnitude of effects revealed that the predicted probability of prime moose kill occurrence increased at a rate of 1 percentile for every 10 m increase in elevation and decreased at a rate of 5 percentiles for every 20% increase in conifer cover. Conversely, the predicted probability of senescent moose kill occurrence increased by 11 percentiles for every 10 m increase in elevation and increased at a rate of 5 percentiles for every 20% increase in conifer cover. Because certain coefficients in regression models were not statistically significant (as indicated by standard errors (se) that overlapped 0 in Fig. 3), we re-ran our models with these coefficients removed. These models produced results that were qualitatively and quantitatively equivalent to those described above for the more fully parameterized models (see Fig. S1).

## Discussion

Here we documented landscape patterns of predation, for wolf-killed moose, that were modulated by prey life history stage. Specifically, the distribution of winter wolf-killed senescent moose was significantly clustered (Fig. 2b) and the core distribution of kill sites was located in a single kill zone in the northeast portion of the island (Fig. 1). Comparatively, prime moose carcasses tended to be more broadly distributed (Fig. 2a) with kill zones distributed among 13 regions of Isle Royale (Fig. 1). Furthermore, the habitat features of kill sites differed substantially between prime and senescent moose (Fig. 3).

On Isle Royale, prime moose were more likely to be killed by wolves in shoreline habitats (Fig. 1). Shoreline habitats on the island represent both riskier environments (because they are used more frequently by wolves) and better foraging opportunities because of the increased prevalence of conifers in those habitats [20]. By contrast, the probability of kill occurrence for senescent moose increased in one zone in the northeast part of Isle Royale at sites characterized by high elevation with patches of dense coniferous forest structure (Figs. 1 and 3). Use of these habitat features is consistent with antipredator behavior of Isle Royale moose [26]. For instance, moose will use dense coniferous forest structure to decrease the probability of detection from wolves and to increase the probability of fending off a wolf attack [67]. Additionally, within these patches of dense coniferous forest structure, moose would have access to forage and would also experience reduced snow depths under the canopy [68]–[70]. This emphasizes that this habitat would have advantages to a moose apart from wolf-avoidance. Thus, senescent moose seem to be killed in patches of improved foraging within a matrix of habitat with comparatively low predation risk.

In a previous analysis we highlighted how the habitat use of prime-aged and senescent-aged moose differed with respect to distance to the Isle Royale shoreline [20]. Specifically, we noted that across a 50-year period senescent-aged moose were wolf-killed on average 200 m closer to the shoreline than prime-aged moose, though this general pattern was greatly affected by relative predation risk and winter severity. Here we identify that kill zones for wolf-killed prime moose tended to be in near-shore habitat whereas the kill zone for senescent moose was centered on inland habitat (Fig. 1). The possible explanations for this apparent discrepancy are: Distance to shore was the response variable in Montgomery et al. [20] whereas here it is one of six habitat features considered as predictor variables. That difference between analyses is relevant because when causal relationships are complicated, as they are for many ecological phenomena, a variable may correlate with a response when considered by itself, but not when other predictors are taken into account [71]. While our previous analysis revealed useful patterns, the present analysis is designed to focus more on the assessment of landscape patterns. For that reason, we used the percentile of the kill zone at kill sites as a response variable to characterize the probability of kill occurrence. Finally, we focused this assessment on the period between 2000 and 2008, corresponding to the vintage of the geospatial data describing the island, whereas Montgomery et al. [20] examined a 50-year period. An important general lesson learned from the extensive Isle Royale wolf-moose project is that the importance of particular patterns and processes tends to vary with time (e.g., [19], [72]).

Underlying these landscape-level processes are exogenous (i.e., habitat heterogeneity ultimately influenced by Pleistocene glaciation; [38] and endogenous processes (i.e., stage-specific space use of moose and stage-specific predation by wolves). Previous work has suggested how habitat heterogeneity and complex prey life histories can lead to patterns in trophic ecology (e.g., [9], [73]). Such patterns however, appear most probable for species with dramatically varied life stages, such as fish, amphibians, and insects. Here we demonstrate that similar processes may also be relevant to terrestrial mammals with relatively subtle changes in life history associated with the transition from prime to senescent condition.

**Figure 4:**
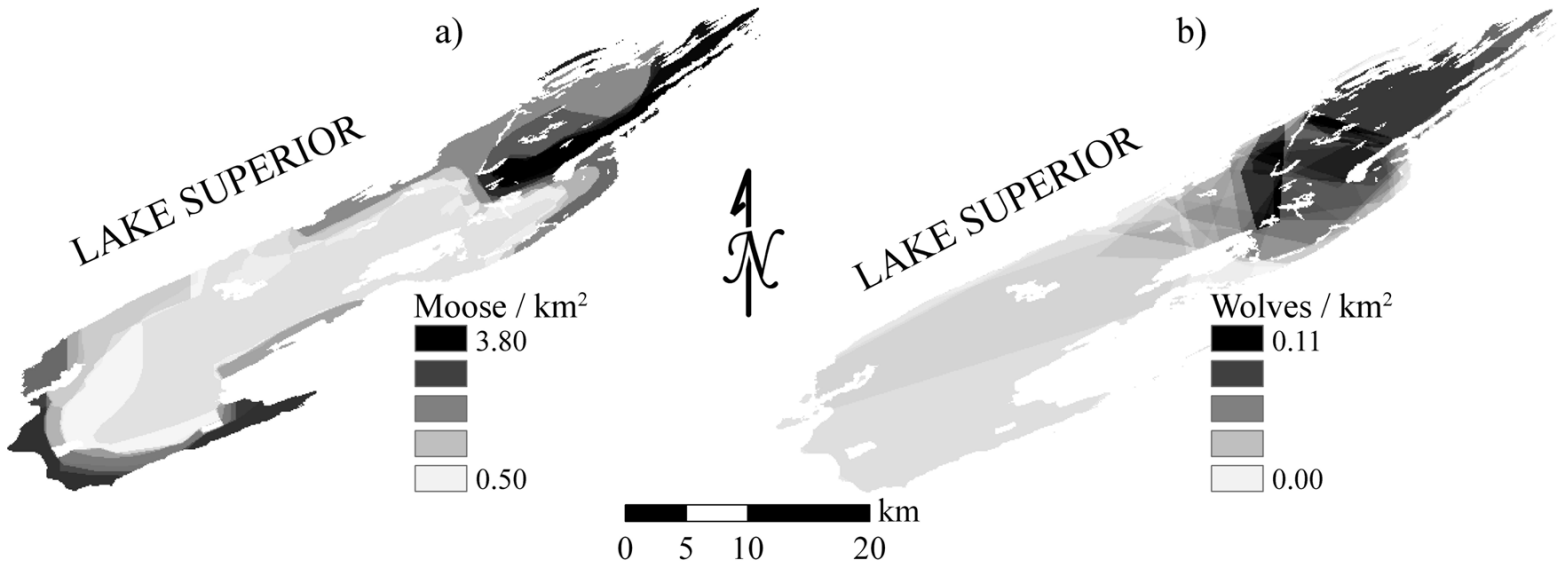
The spatial distribution for a) moose and b) wolves in Isle Royale National Park, Lake Superior, USA. These distributions were documented each year via aerial surveys (e.g., Vucetich and Peterson [43]). Each panel show the means population density, averaged across each year of the study period, 2000–2008.

Landscape patterns in predation (in particular, spatial heterogeneity in kill rates) are one of the basic mechanisms that can result in a ratio-dependent functional response [74], which tend to influence food chain stability and the likelihood of trophic cascades [75],[76]. While the kill rates of Isle Royale wolves are ratio-dependent, it had previously been assumed that the underlying mechanism was interference competition among wolves [39]. The results presented here and the scale-invariant nature of ratio dependency for Isle Royale wolves [77] suggest landscape patterns in predation may also play an important role in shaping the functional response of wolves. In particular, the spatial patterns documented here (Figs. 1 and 4) and elsewhere [20] are known to affect the functional response of wolves, mainly the rate at which they encounter vulnerable prey and prey in preferred age classes preferred [29],[78].

Landscape patterns in predation can also have a destabilizing influence on predation dynamics (*sensu* [79]). This is especially the case when consumers tend to aggregate to portions of a landscape where resources are most dense, and when that aggregation results in positive spatial covariance in the abundance of consumers and resources [80]–[82]. This circumstance seems to characterize the Isle Royale system, in that wolf, moose, and balsam fir density (specifically in near-shore areas) are all greatest in the same portion of the island, the east end (Fig. 4). The destabilizing influence of landscape patterns can also depend on the extent to which spatial heterogeneity across a landscape maximizes the fitness of individual prey [83]. For example, the stability of predator-prey dynamics can be influenced by prey fitness (e.g., *r* in the Lotka-Volterra formulation of predation; see also [84]), which can in turn be influenced by the role of habitat decisions on population mortality rate. Dynamics like this are likely relevant to our results because the observed landscape patterns (Fig. 2) were, at least in part, the result of risk-sensitive foraging decisions of moose, and stage-structured variation in those decisions. Those differences likely arise from prime moose being less vulnerable to predation and more sensitive to fitness-maximizing decisions. In other words, our results represent stage-structured habitat decisions that likely affect fitness, which could alter the stability of predator-prey dynamics. At least, there is reason to expect complicated population dynamics to emerge from the interaction of landscape patterns and stage-structured dynamics [73].

Conversely, spatial heterogeneity across a landscape can have a stabilizing influence on predator-prey dynamics through other potentially relevant mechanisms involving, for example, spatial heterogeneity in density and non-linear responses to density-dependent processes [79]. In general, the influence of spatial heterogeneity on population stability and its ecosystem consequences can be as important as it is difficult to understand because that influence can depend on so many nuances [79],[84]. For decades, wolf-moose dynamics on Isle Royale had been considered too small and consequently too spatially homogenous to expect that explicit consideration of spatial variation would be relevant. After all, Isle Royale is only about three times the size of an average wolf pack territory [85]. While additional analyses are required to conclude that spatial variation is important in this case, recent and accumulating evidence suggests that it may well be [19],[20],[86],[87]. The general lesson is that spatial heterogeneity may be more important for predation dynamics than is at first apparent, particularly for terrestrial mammalian systems.

## Supporting Information

Figure S1 Standardized regression coefficients with standard error bars from the multiple linear regression model (with insignificant coefficients removed) describing habitat features at prime and senescent moose kill sites in Isle Royale National Park, Lake Superior, USA, 2000–2008. The response variable for this model was the percentile of the kernel density estimate (see Fig. 1) at each site where a moose was wolf-killed which depicts the probability of kill occurrence. Distance to shore refers to the Lake Superior shoreline, and distance to lake refers to inland lakes within Isle Royale.
(JPG)
