# Supplementary figures and images for: Where Wolves Kill Moose: The Influence of Prey Life History Dynamics on the Landscape Ecology of Predation
Source: PLoS One. 2014 Mar 12;9(3):e91414. doi: 10.1371/journal.pone.0091414 (PMC3951347; doi:10.1371/journal.pone.0091414)

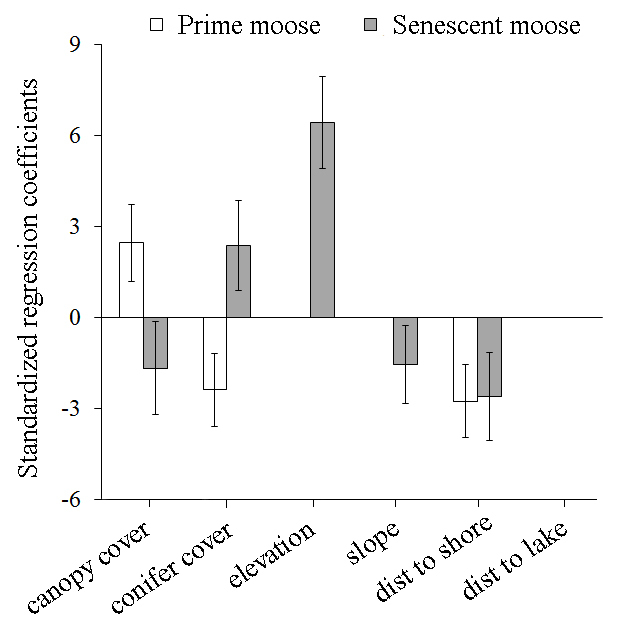

Supplement: Figure S1 — Standardized regression coefficients with standard error bars from the multiple linear regression model (with insignificant coefficients removed) describing habitat features at prime and senescent moose kill sites in Isle Royale National Park, Lake Superior, USA, 2000–2008. The response variable for this model was the percentile of the kernel density estimate (see Fig. 1) at each site where a moose was wolf-killed which depicts the probability of kill occurrence. Distance to shore refers to the Lake Superior shoreline, and distance to lake refers to inland lakes within Isle Royale. (JPG) [file pone.0091414.s001.jpg]
